# T Cell Reactivity against Mycolyl Transferase Antigen 85 of *M. tuberculosis* in HIV-TB Coinfected Subjects and in AIDS Patients Suffering from Tuberculosis and Nontuberculous Mycobacterial Infections

**DOI:** 10.1155/2011/640309

**Published:** 2010-09-27

**Authors:** Pascal Launois, Annie Drowart, Eliane Bourreau, Pierre Couppie, Claire-Michèle Farber, Jean-Paul Van Vooren, Kris Huygen

**Affiliations:** ^1^WHO-IRTC, Department of Biochemistry, University of Lausanne, Chemin des Boveresses, 1066 Epalinges, Switzerland; ^2^WHO/TDR, Avenue Appia 20, 1211 Geneva, Switzerland; ^3^Hôpital Erasme, ULB, 1070 Bruxelles, Belgium; ^4^Chest Department, Centre Hospitalier Universitaire Brugmann, Place Arthur Van Gehuchten 4, 1020 Bruxelles, Belgium; ^5^Immunologie des Leishmanioses, Institut Pasteur de la Guyane, 97306 Cayenne, French Guiana; ^6^Institut Guyanais de Dermatologie Tropicale, E.A. 2188, Centre hospitalier Andrée Rosemon, 97300 Cayenne, French Guiana; ^7^Scientific Service Immunology, O.D. Communicable and Infectious Diseases, WIV-ISP-IPH Site Ukkel, 642 Engelandstraat, 1180 Brussels, Belgium

## Abstract

The mycolyl transferase antigen 85 complex is a major secreted protein family from mycobacterial culture filtrate, demonstrating powerful T cell stimulatory properties in most HIV-negative, tuberculin-positive volunteers with latent *M.tuberculosis* infection and only weak responses in HIV-negative tuberculosis patients. Here, we have analyzed T cell reactivity against PPD and Ag85 in HIV-infected individuals, without or with clinical symptoms of tuberculosis, and in AIDS patients with disease caused by nontuberculous mycobacteria. Whereas responses to PPD were not significantly different in HIV-negative and HIV-positive tuberculin-positive volunteers, responses to Ag85 were significantly decreased in the HIV-positive (CDC-A and CDC-B) group. Tuberculosis patients demonstrated low T cell reactivity against Ag85, irrespective of HIV infection, and finally AIDS patients suffering from NTM infections were completely nonreactive to Ag85. A one-year follow-up of twelve HIV-positive tuberculin-positive individuals indicated a decreased reactivity against Ag85 in patients developing clinical tuberculosis, highlighting the protective potential of this antigen.

## 1. Introduction

*Mycobacterium tuberculosis* (*M. tuberculosis)*, the causative agent of tuberculosis (TB), remains the largest single infectious cause of death globally. Indeed, about one third of the world population is infected with *M. tuberculosis,* and in 2008 an estimated 1.3 million people died of tuberculosis and an estimated 9.3 million people developed the disease worldwide (http://www.who.int/mediacentre/factsheets/fs104/en/index.html). Administration for many decades of the attenuated strain of *M. bovis* BCG (Bacillus Calmette-Guérin) as a vaccine—which in 2000 covered 86% of the world population [1]—has not been able to eradicate this poverty-related sickness. The long duration of treatment with a combination of three to four antibiotics, the lack of compliance, and the unreliable drug supply have been important causes for the emergence of multidrug resistant (MDR) strains of *M. tuberculosis *[2]. Moreover, at least one third of the more than 30 million people infected with the human immunodeficiency virus HIV worldwide are also infected with *M. tuberculosis*. HIV-positive subjects are at increased risk to reactivate a latent *M. tuberculosis *infection, even when CD4^+^ counts are still relatively unaffected. While the risk to reactivate TB is in the order of 10% on a lifetime basis for HIV-negative persons, there is an annual estimated risk of 10% for HIV-*M. tuberculosis *coinfected subjects [3, 4]. In 2008, there were 1.4 million HIV-positive tuberculosis patients globally and 500,000 people died of HIV-associated TB http://www.who.int/tb/challenges/hiv/factsheet_hivtb_2009update.pdf. 

It is clear that the development of improved prophylactic and immunotherapeutic vaccines, which could be administered to both immunocompetent and immunocompromised people, is urgently needed to control the global threat of TB. For this purpose, the identification of major antigens recognized by the protective immune response against *M. tuberculosis* remains an essential step. This has led to the investigation, in preclinical animal models, of more than one-hundred new vaccine candidates [5, 6], of which some have now progressed to phase 1 and phase 2 clinical trials [7–9]. Among the secreted and surface-exposed proteins from the pathogen, important for the elicitation of protective immune responses against TB [10, 11], components of the secreted Ag85 complex, a major protein fraction of all mycobacterial culture filtrates, are among the most promising vaccine candidates [12]. The Ag85 complex is a 30–32 kD family of three proteins (Ag85A, Ag85B, and Ag85C), which all three possess enzymatic mycolyl transferase activity involved in the coupling of mycolic acids to the arabinogalactan of the cell wall and in the biogenesis of cord factor [13, 14]. Ag85 is considered to be a virulence factor as its expression is needed for intracellular survival within macrophages [14]. On the other hand, the Ag85 components are very immunogenic. In mice, guinea pigs, and nonhuman primates, vaccination with members of the Ag85 family was reported to stimulate strong humoral and cell-mediated immune responses and to confer significant protection against challenge with live *M. tuberculosis* H37Rv [15–19]. We have previously reported that the Ag85A protein induces strong T cell proliferation and IFN-*γ* production in most healthy individuals latently infected with *M. tuberculosis*/*M. leprae* [20, 21] and in BCG-vaccinated mice [22] but not in patients suffering from tuberculosis or lepromatous leprosy [23, 24]. Both Ag85A and Ag85B are actually being tested in clinical phase 1 and 2 trials, respectively, as recombinant Modified Vaccinia Virus in a BCG prime-MVA-Ag85A boost protocol [8, 25] or as a subunit fusion protein vaccine coupled to ESAT-6 [7, 26].

The aim of this paper was to evaluate memory T cell responses against Ag85 in a context of HIV infection by measuring proliferative responses and IFN-*γ* secretion upon *in vitro *stimulation of peripheral blood leukocytes. In parallel, T cell responses were also analyzed against crude mycobacterial antigen, that is, purified protein derivative (PPD) and against polyclonal mitogens Phytohemagglutinin (PHA) and Pokeweed Mitogen (PWM), as indicators of overall immune status.

## 2. Materials and Methods

### 2.1. Subjects

#### 2.1.1. Belgian  Study

Twenty-three  HIV-negative/tuberculin-positive subjects were recruited among laboratory workers of the WIV-ISP-IPH and the Hôpital Erasme, Brussels. One subject had received prior BCG vaccination (not performed on a routine basis in Belgium) whereas the others were presenting with a latent *M. tuberculosis *infection or were cured of pulmonary TB patients (three subjects). Fifteen HIV-negative/tuberculin-negative subjects were also recruited among laboratory workers of the WIV-ISP-IPH and the Hôpital Erasme. Seventeen HIV-positive/tuberculin-positive subjects without clinical symptoms of tuberculosis (CDC-A) were recruited at the Hôpital Erasme (mean CD4^+^ counts: 454 ± 228/mm^3^). Ten AIDS patients presenting with clinical signs of active tuberculosis were recruited at the Hôpital Erasme; three suffered from pulmonary TB, five from extrapulmonary TB, and two from pleural effusion (Mean CD4^+^ count: 202 ± 36/mm^3^). Finally seven AIDS patients suffering from disease caused by mycobacteria other than tuberculosis (NTM) (*M. avium-intracellulare* (*n* = 6) or *M. gordonae *(*n* = 1)) were enrolled at the Hôpital Saint-Pierre in Brussels (Mean CD4^+^ count: 11/mm^3^).

#### 2.1.2. French Guyana Study

Seven tuberculin-positive and 6 tuberculin-negative HIV-negative controls without clinical symptoms of tuberculosis were enrolled in the study. None of them had suffered from active tuberculosis prior the study, and the X-ray was normal for all of them. A total of forty-five HIV-positive subjects without clinical symptoms of tuberculosis were analyzed. Twenty-eight HIV-positive subjects were classified in the category A of the CDC classification (without clinical symptoms of tuberculosis); seventeen were tuberculin-positive and eleven were tuberculin-negative. Seventeen HIV-positive subjects classified in the category B of the CDC classification, suffering from either varicella zoster or/and candidiasis (which are the most frequent opportunistic infections in HIV-positive patients in French Guyana), were also studied. Among them seven were tuberculin positive and ten were tuberculin negative. Finally, fourteen adult patients with active pulmonary tuberculosis before multidrug therapy were monitored at the Centre Hospitalier Andrée Rosemon in Cayenne, French Guyana. Six of these patients were HIV negative, and 8 were HIV positive. All HIV negative, and seven HIV positive patients were diagnosed with pulmonary tuberculosis whereas one HIV-positive patient was diagnosed with extrapulmonary TB. All subjects had been vaccinated with BCG at birth as currently done in French Guyana. Informed consent was obtained from the subjects, and the human guidelines given by the Comité Consultatif de Protection des Personnes dans la Recherche Médicale (CCPRB) from Guadeloupe were followed.

### 2.2. Tuberculin Test

Subjects were injected intradermally with 0.1 mL of tuberculin (10 units, Tubertest, Sanofi Pasteur) on the forearm. Reactions were determined by measuring transverse diameter of induration 48/72 h after administration. For HIV-positive subjects, an induration of more than 5 mm in diameter was considered as positive. For HIV-negative subjects, an induration of more than 10 mm was considered as positive.

### 2.3. Antigens

*M. bovis *BCG (strain GL2) was grown for 2 weeks as a surface pellicle on synthetic Sauton medium. BCG culture filtrate (CF) was concentrated by ammonium-sulfate precipitation (70% saturation), dialyzed against PBS, sterilized by filtration, and stored at −20° as previously described [22]. CF was used at a final concentration of 5 *μ*g/mL. Purified protein derivate from *M. tuberculosis* (PPD) (Statens Serum Institute, Copenhagen, Denmark) was used at the concentration of 5 *μ*g/mL (IFN-*γ*  production) or 25 *μ*g/mL (lymphoproliferative assays). Native Ag85 complex was purified from BCG culture filtrate by sequential chromatography on phenyl-Sepharose and DEAE-Sephacel (Pharmacia, Uppsala, Sweden) as previously described and used at a final concentration of 10 *μ*g/mL [27]. Phytohemagglutinin and Pokeweed Mitogen (both from Sigma, L'Isle d'Abeau, France) were used as polyclonal T cell and T cell-dependent B cell mitogen, respectively.

### 2.4. Lymphoproliferation Assays (Brussels Study)

Heparinized whole blood, collected by venipuncture, was diluted 1 : 10 in RPMI-1640 medium, supplemented with HEPES, L-glutamine, penicillin/streptomycin, and 5 × 10^−5^ M 2-mercapto-ethanol. Cells were cultured in round bottom microwell plates (Greiner) in a humidified CO_2_ incubator at 37° for 7 days. Tritiated thymidine (Amersham) was added to the cells during the last 20 hours of culture. Cells were harvested on a Skatron Cell Harvester, and filters were counted in a Beckman LS Betaplate scintillation counter. Mean counts per minute (cpm) were calculated from quadruplicate cultures.

### 2.5. IFN-*γ* Production and IFN-*γ* Detection (Cayenne Study)

PBMCs were obtained by venipuncture, isolated on Ficoll-Hypaque gradient (*d* = 1,077), and suspended in RPMI medium supplemented with 2 mM L-glutamine, 100 U of penicillin/mL, 0.1 mg of streptomycin/mL (all from Sigma), and 10% human heat-inactivated serum from red blood cell group AB. Cells were plated at 10^6^/mL in flat-bottom 24-well plates with or without antigens. The culture supernatants were harvested after 7 days and stored at −20°C. IFN-*γ* levels were measured using specific IFN-*γ* sandwich ELISA (sensitivity of 10 pg/mL) using NIB42 clone (mouse IgG1) as capture antibody and 4SB2 clone (mouse IgG1) as detection antibody (both from Pharmingen San Diego, CA).

### 2.6. Statistical Analysis

Student's *t*-test was used for statistical evaluation. Statistical analysis was done on log _10_ values of IFN-*γ* titers, and IFN-*γ* titers below 10 pg/mL were considered as 0.1 log _10_ for statistical calculations. Proliferative responses were analyzed using GraphPad Prism 4 software.

## 3. Results

### 3.1. Study Participants

Table 1 shows a summary of the different participant groups from Belgium (Brussels study) and French Guyana (Cayenne study) analyzed in this study. All work was conducted in accordance with the Declaration of Helsinki. Experiments were performed with the understanding and the consent of the human subjects and with approval of the two local Ethical Committees.

### 3.2. Lymphoproliferative Responses of HIV-Negative and HIV-Positive Subjects (Brussels Study)

**Figure 1:**
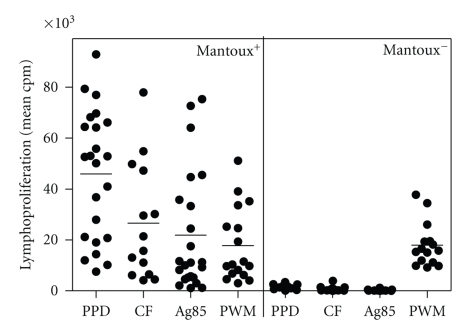
Proliferative responses (mean cpm) in response to PPD, CF from BCG, purified Ag85, and Pokeweed Mitogen in healthy tuberculin-positive (*n* = 23) and tuberculin-negative HIV-negative (*n* = 15) volunteers, as measured after 7 days of culture, using a whole blood assay (heparinized blood diluted 1 : 10).

In order to determine threshold values for positivity, 23 healthy tuberculin-positive and 15 tuberculin-negative subjects (all HIV-negative) were tested for their *in vitro *lymphoproliferative response using a diluted whole blood assay. As shown in Figure 1, proliferative responses upon stimulation with PPD, culture filtrate from BCG, and purified Ag85 complex from BCG culture filtrate were significantly higher in the tuberculin-positive (Mantoux^+^) than in the tuberculin-negative (Mantoux^−^) group (*P* < .01), whereas no difference was observed following stimulation with polyclonal PWM (*P* = .936). 

**Figure 2:**
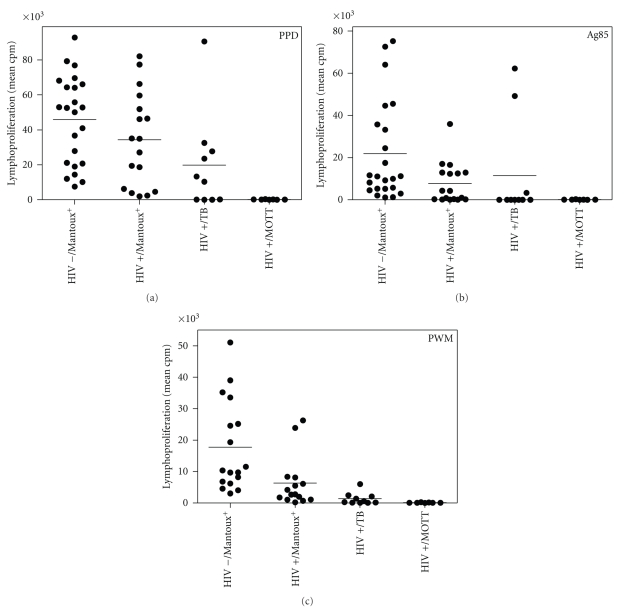
Lymphoproliferative response against PPD, Ag85, and PWM in HIV-negative/tuberculin-positive, HIV-positive/tuberculin-positive, HIV-positive patients with TB and HIV-positive patients with MOTT disease. Proliferative responses (mean cpm of quadruplicate cultures) as measured after 7 days of culture, using a whole blood assay (heparinized blood diluted 1 : 10).

Next, responses in these HIV-negative, tuberculin-positive subjects were also compared to proliferative responses in asymptomatic HIV-positive subjects latently infected with *M.tuberculosis *(TST >5 mm), in ten AIDS patients suffering from active tuberculosis, and in seven AIDS patients suffering from disease caused by mycobacteria other than tuberculosis (MOTT/NTM). *In vitro *proliferative responses to PPD of HIV-negative and HIV-positive subjects with a positive tuberculin skin test were not significantly different between the two groups (mean ± SEM: 45,940 ± 5,255 (*n* = 23) cfr 34,220 ± 6,571 (*n* = 17) *P* = .167) (Figure 2(a)). Responses to CF were not significantly different either (data not shown). In contrast, *in vitro *proliferative responses to purified Ag85 (Figure 2(b)) were significantly higher in the HIV-negative than in the HIV-positive group (21,870 ± 4,904 (*n* = 23) cfr 7,775 ± 2,360 (*n* = 17), *P* < .05). Using a cutoff value of 907 cpm (mean + 2 SD values of Ag85-specific responses of 15 HIV-negative/tuberculin-negative volunteers, Figure 1), only 10/17 (59%) of the HIV-positive subjects demonstrated a positive proliferative T cell response to Ag85. Likewise, responses to the polyclonal mitogen PWM (Figure 2(c)) were also significantly higher in the HIV-negative group than in the HIV-positive group (17,780 ± 3,520 (*n* = 17) cfr 6,330 ± 2,078 (*n* = 15), *P* < .05). 

Finally, lymphoproliferative responses were also examined in AIDS patients with clinical signs of active tuberculosis or NTM (MOTT) disease. Using a cutoff value of 3,627 cpm (mean + 2 SD values of PPD-specific responses of 15 HIV-negative/tuberculin-negative healthy volunteers), positive responses to PPD could be detected in 6/10 (60%) of HIV-positive tuberculosis patients (19,860 ± 8,779) (Figure 2(a)). Proliferative responses against Ag85 (Figure 2(b)) were much more dramatically affected and positive only in two patients (actually the two patients suffering from pleural effusion) (mean (*n* = 10): 11,520 ± 7,444; mean (*n* = 8) 485 ± 408). Responses to PWM (Figure 2(c)) were finally also strongly depressed in 9/10 patients (1,334 ± 593). 

Reflecting their dramatic decrease in CD4^+^ counts (mean: 11/mm^3^), AIDS patients diagnosed with NTM (MOTT) disease were completely unreactive to all antigens tested (PPD: 147 ± 41; Ag85: 105 ± 32; PWM 104 ± 31) (Figures 2(a), 2(b), and 2(c), resp.).

### 3.3. IFN-*γ* Responses of HIV-Negative and HIV-Positive (CDC-A) Subjects (Cayenne Study)

*In vitro *IFN-*γ* production was analyzed in Cayenne in a 7-day cultured supernatant of purified PBMC from 13 HIV-negative controls with either a positive (*n* = 7) or a negative (*n* = 6) tuberculin skin test. Confirming the proliferative responses, purified PBMC from tuberculin-positive controls produced statistically higher IFN-*γ* levels than tuberculin-negative controls for all the mycobacterial antigens tested. (Table 2, *P* < .05). On the other hand, IFN-*γ* levels in response to PHA were not statistically different in these two groups of HIV-negative controls. 

Next, IFN-*γ* production was analyzed in 28 HIV-positive controls without clinical symptoms of tuberculosis. Seventeen were tuberculin positive, and eleven were tuberculin negative. HIV-positive/tuberculin-positive subjects produced significantly more IFN-*γ* than HIV-positive/tuberculin-negative subjects upon stimulation with CF, Ag85, or PPD, but not upon stimulation with PHA or PWM (Table 2). The number of CD4^+^ T cells was not statistically different between both groups, that is, 498 ± 264/mm^3^ in tuberculin-positive and 467 ± 286/mm^3^ in tuberculin-negative subjects, respectively.

Mean IFN-*γ* production in response to all mycobacterial antigens tended to be higher in HIV-negative than in HIV-positive skin test-positive persons (Table 2), but (in contrast to the lymphoproliferative responses) this difference was only significant in response to PPD.

### 3.4. IFN-*γ* Responses of PBMC Recovered from HIV-Negative and HIV-Positive Subjects with Clinical Symptoms of Tuberculosis

**Figure 3:**
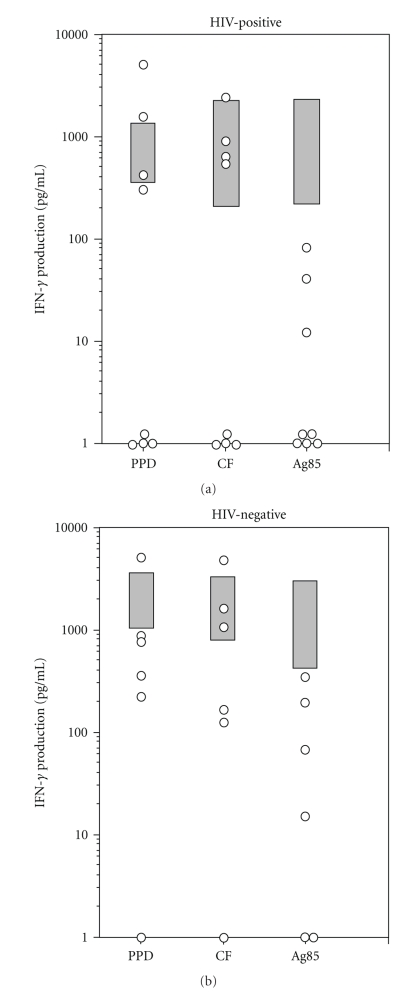
Individual IFN-*γ* production (pg/mL) by PBMC in response to different antigens from HIV-positive (a) or HIV-negative (b) patients with active tuberculosis. A negative response was considered as less than mean −2SD of the IFN-*γ* production by PBMC from tuberculin-positive/HIV-positive (a) and tuberculin-positive/HIV-negative (b) controls. The circle patients with active tuberculosis. The grey rectangle mean ± 2SD of the IFN-*γ* produced by PBMC from tuberculin-positive/HIV-positive or HIV-negative controls.

IFN-*γ* production in response to different mycobacterial antigens was also analyzed in HIV-positive (*n* = 8) and HIV-negative (*n* = 6) patients suffering from active tuberculosis. A cutoff level for negative IFN-*γ* production to each antigen was determined as less than the mean minus two 2SD values of the IFN-*γ* level measured in tuberculin-positive, HIV-positive, respectively, HIV-negative controls without symptoms of tuberculosis (indicated by the grey bars, calculated from results of Table 2). Using this threshold, 3 (50%) and five (83%) out of 6 HIV-negative patients with active tuberculosis were unresponsive to CF and PPD, respectively (Figure 3(b)). Confirming our previous findings on larger groups of TB patients [20, 23], IFN-*γ* responses to Ag85 were very low in HIV-negative patients with active tuberculosis, and in this study actually all 6 were unresponsive to Ag85. In the group of 8 HIV-positive patients with active tuberculosis, 4 (50%) and 5 (66%) were unresponsive to CF and PPD, respectively. All of these 8 patients were unresponsive to Ag85 (Figure 3(a)). Both HIV-negative and HIV-positive subjects with active tuberculosis responded to PWM and PHA even if the IFN-*γ* titers in response to PHA were lower in HIV-positive than in HIV-negative tuberculosis patients (1940 ± 1689 pg/mL versus 4161 ± 2123 pg/mL in response to PHA; 3550 ± 1906 pg/mL versus 2625 ± 480 pg/mL in response to PWM). CD4^+^ T cell counts in HIV-positive patients with active tuberculosis ranged from 97/mm^3^ to 740/mm^3^ (mean: 384 ± 277/mm^3^). Since all the HIV-positive (and negative) patients with tuberculosis were unresponsive to Ag85, their inability to react with this antigen was clearly not related to the CD4^+^ T cell count. Altogether these results confirm the notion that the inability to produce IFN-*γ* in response to Ag85 is a characteristic of active tuberculosis, irrespective of HIV coinfection [23].

### 3.5. IFN-*γ* Response to Ag85 in HIV-Positive Patients with Varicella Zoster and/or Candida Infections (CDC-B)

**Figure 4:**
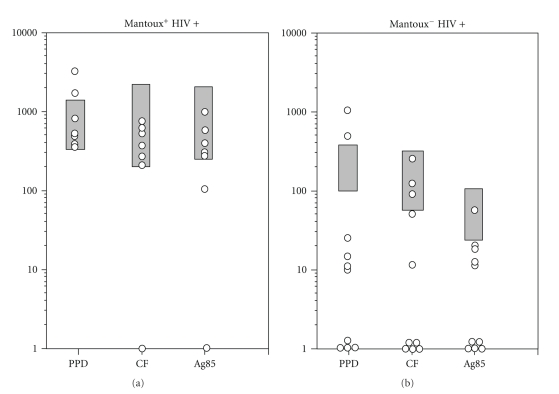
Individual IFN-*γ* production (pg/mL) in PBMC in response to different antigens from tuberculin-positive (a) or tuberculin-negative (b) HIV-positive CDC-B subjects suffering from varicella zoster and/or candidiasis. A negative response was considered as less than mean −2SD of the IFN-*γ* production by PBMC from healthy tuberculin-positive (a) and tuberculin-negative (b) HIV-positive controls. The circle: subjects with varicella zoster and/or candidiasis (category B of the CDC classification). The grey rectangle: mean ± 2SD of the IFN-*γ* produced by PBMC from tuberculin-positive (a) and tuberculin-negative (b) HIV-positive controls.

IFN-*γ* responses to Ag85 were also examined in tuberculin-positive and tuberculin-negative HIV-positive patients with varicella zoster or candida infections (CDC-B) that are the most common opportunistic infections observed in HIV patients in French Guyana. Among the 10 tuberculin-negative/HIV-positive CDC-B subjects, 9 (90%) were unresponsive to Ag85. Furthermore, seven (70%) were unresponsive to CF, and 8 (80%) to PPD (Figure 4(b)). Among the 7 tuberculin-positive/HIV-positive CDC-B subjects, 2 (29%) were unresponsive to Ag85. On the other hand, all were responders to PPD and CF (Figure 4(a)). IFN-*γ* responses to PHA were not different in tuberculin-positive from tuberculin-negative HIV-positive persons suffering from varicella zoster and/or candidiasis (1471 ± 1104 and 1881 ± 1196 pg/mL, resp.). Furthermore, even if the CD4^+^ T cell counts were lower in the tuberculin-negative group (272 ± 190/mm^3^) than in the tuberculin-positive group (400 ± 146/mm^3^), this difference was not significant. The unresponsiveness to Ag85 in 2/7 of these tuberculin-positive subjects in the absence of clinical signs of tuberculosis was suggestive of a subclinical infection with mycobacteria. To test this hypothesis, the 7 patients were reexamined one year later. During this year, two of them developed a biologically confirmed infection with mycobacteria, one with *M. tuberculosis* and one with *M. fortuitum*. Interestingly, these two patients were precisely those who were unresponsive to Ag85 the previous year.

### 3.6. Development of Tuberculosis in Tuberculin-Positive HIV-Positive Subjects (CDC-A) Is Associated with Decreased Ag85-Specific Responses

**Figure 5:**
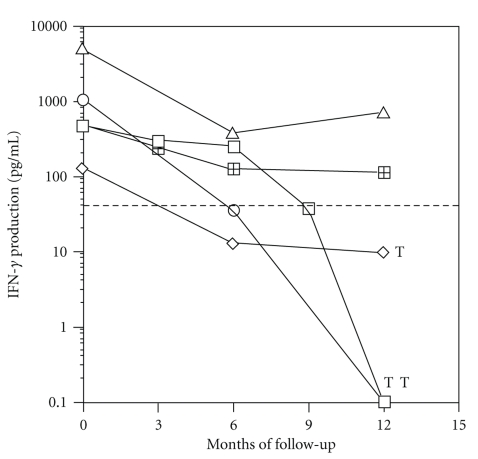
Evolution of Ag85-specific IFN-*γ* response in 5 HIV-positive latently infected subjects. Individual IFN-*γ* production (pg/mL) of PBMC in response to Ag85 from 5 tuberculin-positive/HIV-positive subjects at baseline *t* = 0 and 3, 6, 9, and 12 months later. T = diagnosis of active tuberculosis. Dashed line: mean of IFN-*γ* production minus 2SD in tuberculin negative/HIV positive subjects.

Five tuberculin-positive/HIV-positive subjects (CDC-A) were examined at month 0, 3, 6, 9, and 12 for their IFN-*γ* production in response to Ag85 and for clinical signs of tuberculosis. As shown in Figure 5, all five subjects mounted a positive IFN-*γ* response to Ag85 at the beginning of the followup. Two out of five subjects maintained their responsiveness to Ag85 and remained free of clinical symptoms. In contrast, one subject showed a moderate and two subjects showed a dramatic decrease in IFN-*γ* in response to Ag85, and all three presented with signs of clinical tuberculosis at the end of the 12-month follow-up period.

## 4. Discussion

A major challenge in tuberculosis control is a better understanding of latent *M. tuberculosis* infection. Indeed, many adult TB cases result from the reactivation of an initially controlled latent *M. tuberculosis* infection. A defective immune system, caused by iatrogenic immunosuppression, poverty-related malnutrition, stress, ageing, and genetic factors provide the basis of this reactivation. Infection with the human immunodeficiency virus is another very important risk factor for developing clinical tuberculosis, particularly in third-world countries of the South. It is estimated that more than one third of HIV-positive individuals are coinfected with *M. tuberculosis, *and approximately 12% of AIDS deaths are due to TB [3, 28]. *M. tuberculosis *reactivation in HIV-coinfected subjects is generally observed at a 65 to 75% drop in CD4^+^ T cell counts, which is actually much earlier than for the classical opportunistic infections caused by mycobacteria other than tuberculosis, such as bacteria of the *M. avium-intracellulare *complex*.*

Even though tuberculosis preventive therapies can offer a short-term effect in reducing the incidence of TB for HIV-infected adults, they are not effective in delaying HIV disease progression to AIDS. Thus, in a randomized controlled trial of 1053 HIV-positive Zambian adults receiving isoniazid for 6 months or rifampicin plus pyrazinamide for 3 months, both preventive treatment regimens protected against tuberculosis for at least 2.5 years but appeared to have no effect on HIV progression or mortality [29]. Similar findings were reported in a randomized placebo-controlled trial in Kampala, Uganda on 2,736 PPD-positive and anergic HIV-infected adults treated with isoniazid (INH) for 6 months, INH plus rifampicin for 3 months, or INH plus rifampicin plus pyrazinamide for 3 months [30, 31].

Here, we have analyzed mycobacteria-specific T cell reactivity in *M. tuberculosis*-infected HIV-positive subjects at different stages of HIV progression. We observed that lymphoproliferative memory T cell responses to the purified mycolyl transferase Ag85 are impaired very early during HIV infection at moments when T cell responses to crude PPD and culture filtrate are still within the normal range found in tuberculin-positive/HIV-negative healthy volunteers. Positive proliferative responses against Ag85 could be detected in only 59% of the HIV-positive subjects, presenting with a mean CD4^+^ count of more than 450/mm^3^. In contrast, average Ag85-specific IFN-*γ* response in asymptomatic tuberculin-positive subjects was not significantly different in the group of HIV-negative and HIV-positive subjects (although there was a tendency to decrease) but interestingly a follow-up analysis of these Ag85-specific IFN-*γ* responses in five tuberculin-positive subjects classified as CDC-A according to CDC classification [32] over a period of one year indicated that decreased reactivity against Ag85 in three of them coincided with reactivation and development of clinical tuberculosis. Likewise, a followup in the group of the seven tuberculin-positive subjects classified as CDC-B showed development of clinical disease precisely in the two subjects with initial low IFN-*γ* reactivity to Ag85. Despite the limited number of subjects in this followup, our results strongly suggest a protective role of Ag85-specific T cell responses in the control of *M. tuberculosis* infection. 

Vaccination with live attenuated BCG is not recommended in immunocompromised individuals. However, boosting of the low Ag85-specific responses in these patients using subunit vaccination could be an alternative, particularly in patients with restored CD4^+^ T cell counts after highly active antiretroviral therapy [33–35]. We have previously demonstrated the feasibility of this approach in a preclinical model of CD4 gene knockout mice, partially reconstituted with CD4^+^ T cells and vaccinated with plasmid DNA encoding Ag85A and Ag85B [36].

The tuberculin skin test lacks sensitivity, particularly in HIV-infected individuals and has poor specificity because of antigenic cross-reactivity of PPD with environmental mycobacteria and the BCG vaccine. Only few studies in immunocompromised subjects have used *in vitro *T cell-based assays using purified mycobacterial antigens. Silveira et al. reported on cell-mediated immune responses to five purified mycobacterial antigens in Portuguese HIV-positive and HIV-negative patients with pulmonary tuberculosis [37]. Similar to our results, T cell responses to purified 30 kD protein (the Ag85B component of the Ag85 complex) were lower in the tuberculosis patients than in HIV-negative PPD-positive healthy volunteers, irrespective of HIV infection. Proliferative responses were more strongly affected than IFN-*γ* secretion in that study as well. In Zambia, a highly endemic country for tuberculosis, *ex vivo *IFN-*γ* ELISPOT to *M. tuberculosis-*specific ESAT-6/CFP-10 proteins was found to be more specific and possibly more sensitive than PPD-based methods of detecting latent *M. tuberculosis *infection in HIV-positive subjects [38]. Lymphoproliferative responses and IFN-*γ* secretion to mycobacterial antigens ESAT-6 and Ag85 were also studied in subjects participating in a phase III randomized placebo-controlled trial of a BCG prime-boost vaccine strategy (using whole inactivated *Mycobacterium vaccae*) in Dar es Salaam, Tanzania [39]. Tanzanian HIV-infected adults with CD4^+^ counts > 200/mm^3^ and primed with BCG vaccine in childhood were analyzed. Among 1885 subjects screened, 635 (35%) were classified to have latent TB (as indicated by a PPD skin test of >5 mm) and 13 had active tuberculosis. Subjects with latent TB were more likely to have a T cell response than TB patients. Proliferative response to Ag85 was detected in only 18.7% of these latently infected HIV-infected subjects [40]. In the Brussels study, 10/17 (59%) of the tuberculin-positive/HIV-positive subjects demonstrated a positive proliferative response to Ag85. With respect to Ag85-specific IFN-*γ* production, the Tanzanian study found only 38,6% of latently infected subjects to react to the antigen [41], whereas in our study all seventeen tuberculin-positive, HIV-positive (CDC-A) subjects from Cayenne were found to be reactive. The reason for this lower reactivity levels in the Tanzanian study is not clear. Besides the far smaller number of subjects in our study, another factor may have been the low (0,5 mcg/mL) antigen concentration used in the Tanzanian study. Also, except for one person, the subjects enrolled in our proliferative study had not been vaccinated with BCG, whereas all subjects in the Tanzanian study had been vaccinated with BCG.

## 5. Conclusion

We have demonstrated that Ag85-specific proliferative T cell responses are decreased during *M. tuberculosis*-HIV-coinfection, even in 40% of patients classified as CDC-A and with a positive tuberculin skin test. Furthermore, a one-year follow-up of twelve HIV-positive/tuberculin-positive subjects indicated that decreasing IFN-*γ* reactivity against Ag85 was associated with development of clinical tuberculosis, highlighting the protective potential of this antigen. It would be interesting to monitor Ag85-specific proliferative IFN-*γ* T cell responses in HIV-infected subjects latently infected with *M. tuberculosis *and treated preventively with isoniazid to see whether Ag85-specific responses could be stabilized. Attempting to stabilize these T cell responses by postexposure vaccination might be an alternative. In this context, a proof-of-concept Phase IIb clinical trial to evaluate the protective efficacy of a booster MVA85A vaccination administered to healthy HIV-infected adults in South Africa, Senegal, and The Gambia was started in February 2010 (EDCTP Annual report 2009).

## Figures and Tables

**Table 1:** Study participants.

		Brussels	Cayenne
HIV−	Tuberculin −	15	6
	Tuberculin +	23	7
	Tuberculosis	—	6

HIV+	CDC-A		
	tuberculin −	—	11
	tuberculin+	17	17
	CDC-B		
	tuberculin −	—	10
	tuberculin +	—	7
	Tuberculosis		
	Pulmonary	3	7
	Extrapulmonary	5	1
	Effusion	2	—
	NTM infection	7	—

Number of subjects tested in each group in the two locations.

**Table 2:** IFN-*γ* production of PBMC from tuberculin-positive and tuberculin-negative, HIV-negative and HIV-positive subjects.

	HIV negative		HIV positive (CDC-A)	
Stimulus	tuberculin +	tuberculin –	tuberculin +	tuberculin –
	(7)	(6)	(17)	(11)
PPD	2417 ± 662^∗,°^	157 ± 56	852 ± 369**	238 ± 70
CF	1988 ± 576*	86 ± 28	1210 ± 503**	176 ± 63
Ag85	1644 ± 672*	26 ± 12	1141 ± 456**	84 ± 20
PHA	2635 ± 845	2885 ± 525	3281 ± 1842	2637 ± 1355

IFN-*γ* production in day 7 culture supernatants from purified PBMC. Results are expressed as mean ± standard deviation (pg/mL).

*Significantly different from healthy tuberculin-negative/HIV-negative controls (*P* < .05).

**Significantly different from healthy tuberculin-negative/HIV-positive controls (*P* < .05).

°Significantly different from healthy tuberculin-positive/HIV-positive controls (*P* < .05).
